# Perceptions of Environmental Supports for Physical Activity in African American and White Adults in a Rural County in South Carolina

**Published:** 2005-09-15

**Authors:** Steven P Hooker, Dawn K Wilson, Sarah F Griffin, Barbara E Ainsworth

**Affiliations:** Prevention Research Center, Arnold School of Public Health, University of South Carolina; Department of Psychology, University of South Carolina, Columbia, SC; Prevention Research Center, Arnold School of Public Health, University of South Carolina, Columbia, SC; Department of Exercise and Nutritional Sciences, San Diego State University, San Diego, Calif

## Abstract

**Introduction:**

This study examined the association between perceptions of social and safety-related environmental attributes and physical activity (PA) and walking in African American and white adults.

**Methods:**

In a random-digit–dial telephone survey, 1165 adults in a rural county in South Carolina answered questions about their perceptions of social and safety-related environmental supports for PA and their overall PA and walking behavior. Social perceptions included whether neighbors could be trusted or were perceived to be physically active. Safety-related perceptions included neighborhood safety, the safety of public recreation facilities, problems with unattended dogs, traffic volume, and streetlight quality. Logistic regression models were used to examine the associations between environmental supports and PA and walking stratified by race.

**Results:**

No association between perceived neighborhood environmental supports and PA or walking was observed in African Americans. Among whites, individuals who perceived their neighbors as active were twice (95% confidence interval [CI], 1.19–3.25) as likely to report meeting the recommendation for PA compared with individuals who did not report their neighbors as active. Whites who perceived their neighbors as active were 2.5 times (95% CI, 1.54–4.08) as likely to report meeting the recommendations for walking than whites who did not, and whites who perceived their neighborhoods as safe were 1.8 times (95% CI, 1.03–3.12) as likely to report meeting the recommendations for walking than whites who did not.

**Conclusion:**

These data indicate that perceptions of certain social and safety-related environmental supports were strongly associated with meeting the recommendations for PA and walking among white but not African American adults.

## Introduction

It is firmly established that regular physical activity (PA) reduces the risk of many chronic diseases and increases longevity (1). A joint statement by the Centers for Disease Control and Prevention (CDC) and American College of Sports Medicine concluded that moderate-intensity PA performed for 30 minutes on most days of the week will confer significant physical and mental health benefits (2). Despite the positive relationship between PA and several health outcomes, recent national studies indicate that more than 50% of the U.S. adult population does not achieve recommended PA levels and that white adults have a higher rate of regular PA than African American adults (3). The disparity in rates of PA may then contribute to the health disparities that exist between white and African American adults. This idea has led to an increased interest in gaining a greater understanding about the determinants and mediating factors of PA behaviors, including among racial and ethnic populations (4-6).

The ecological model for health promotion emphasizes multiple levels of influence upon individual behavior — intrapersonal, interpersonal and social, organizational, institutional, community, and policy (7,8). Expanded views of this model include environmental attributes that may play an important role in shaping health behaviors such as PA (9). Physical environmental attributes such as traffic, distance, sidewalks, and aesthetics are examples of factors that may influence PA. Social environmental attributes of interest include trust in neighbors, community norms of PA, and social networks. To gain a better understanding in these emerging areas of interest, research has increasingly emphasized the evaluation of individuals' *perceptions* of social and physical environmental attributes that may support or hinder their PA behavior. There is also speculation that perceptions of different sets of social and physical environmental attributes may influence the PA behavior of different individuals, including people of varying sex, age, race, and ethnicity (10). 

A number of investigators have conducted qualitative studies (i.e., focus groups) to further understanding of perceptions about social and safety-related environmental supports for PA among racial and ethnic populations (11-17). These studies consistently report safety-related environmental features to be influential on PA, including concerns about unsafe facilities, stray dogs, crime, and motorized traffic. In addition, a common theme expressed by people in these studies pertains to social environmental factors: community or neighborhood social cohesion, group participation, and support from family, friends, and neighbors.

Research reporting on the associations between social and safety-related environmental supports and overall PA is rapidly accumulating (18-21). However, relatively few studies have documented the association between such supports and walking (22-27) and even fewer have included more than one racial or ethnic population (22,28-30). The purpose of the present study was to examine the relationship between race and perceptions of social and safety-related environmental supports for recommended levels of PA and walking in white and African American adult residents of a rural county in the southeastern United States.

## Methods

### Participants

The research protocol for the study was reviewed and approved by the University of South Carolina Human Subjects Committee. The detailed random-digit–dial methods for this study have been described previously (22). In summary, residents of a rural county in South Carolina (n = 1270; adults aged 18 to 96 years) were surveyed during January and February 2001. The participants interviewed for this study were selected from a stratified random sample of households with listed telephone numbers. A number of residents proportional to the total population and racial distribution of the population were randomly selected from census tracts to guarantee a balance in the racial profile and the geographic distribution of the study sample. Race and ethnicity were categorized based on the respondent's answer to the following question: "What is your race . . . white, black/African American, Asian/Pacific Islander, American Indian, other, or don't know/not sure?" During the interview, once a household member was contacted, a respondent aged 18 years or older was randomly selected from all of the adults (aged 18 years or older) living in the household using the next-birthday method. That is, when more than one adult was in the household, the initial contact was asked to identify the person with the next birthday, and that person was interviewed. Twenty-one census tracts were surveyed with 2 to 80 respondents per tract (median = 61 per tract).

### Questionnaire on perceptions of social and safety-related environmental supports

Items for the questionnaire were developed from an extensive literature review (9), expert input, and focus groups conducted with residents living in the county where this study took place (13). Respondents provided their home address, length of residency, age, race, sex, height, weight, education level, and income level. Respondents completed seven items on neighborhood-level PA supports. Items on safety-related environmental supports for PA included assessing the perceived volume of traffic in neighborhood, streetlight quality, problems with unattended dogs, the safety of public recreational facilities, and overall neighborhood safety. Items on social environmental supports for PA included assessing the perceived trust of neighbors and the PA level of neighbors. A Likert-type scale was used to assess the social and safety-related environmental supports for PA, with the lower value indicating stronger endorsement. Respondents were told that neighborhood was defined as the area within one half-mile or a 10-minute walk from their home.

The test–retest reliability of these measures ranges between r = 0.42 and r = 0.73 at the neighborhood level (31). Kappa coefficients have demonstrated modest agreement between selected objective indices and self-perception questions for these neighborhood items (31).

### Physical activity measures

The PA module of the 2001 Behavioral Risk Factor Surveillance System (BRFSS) was used to measure PA (3). PA was categorized as meeting the CDC recommendation or not meeting it. The CDC recommendation is 30 minutes or more per day for 5 days or more per week of moderate-intensity PA or 20 minutes or more per session for 3 days or more per week of vigorous-intensity PA (3). The respondents' daily walking behavior was assessed with three additional questions. Respondents were asked if they walked for at least 10 minutes at a time for recreation, exercise, or transportation or while at work. Persons who responded affirmatively were asked how many days per week and how much time per day they walked. From these data, respondents were categorized as walking 150 or more minutes per week (at least 30 minutes per day on at least 5 days per week) or as not walking at least 150 minutes per week.

### Data analyses

Because the sampling rates varied by race, analysis weights were constructed so that results could be generalized to the county population. These weights were incorporated in all descriptive and inferential statistical analyses using SUDAAN 8.0 (Research Triangle Institute, Research Triangle Park, NC).

The primary analyses used logistic regression to account for the two levels of dependent variables and were stratified by race. If respondents answered that they did not know about a given perception or that they did not have a public recreation facility in their neighborhood, they were not included in the analyses for that factor. Respondents who met the CDC recommendation were compared with respondents who did not meet the recommendation. For walking behavior, respondents who were regular walkers (at least 150 minutes per week) were compared with respondents who were irregular walkers (including nonwalkers). An odds ratio greater than unity reflects an increased likelihood of PA or walking at the recommended level. For all regression analyses, education, age, and sex were entered in the model.

## Results

### Demographic and baseline characteristics 

A widely accepted method for determining a survey response rate has been established by the Council of American Survey Research Organizations (CASRO) and the American Association for Public Opinion Research (32). The method employs the following calculation: Survey response rate = (Completed interviews + Partially completed interviews)/(Completed interviews + Partially completed interviews + Refusals + Language barrier + Ill or senile + Consistent answering machines + Unable to complete during fielding period).

Based on this method, a total of 1270 respondents participated in the survey, resulting in a response rate of 54%. Thirty people (2%) who self-reported their race as something other than white or black/African American were excluded from the analyses for this project. Of the 1240 remaining respondents, 1165 (94%) provided complete data for all variables of interest and were included in the final sample. The proportion of African American (41%) and white (59%) adults in the final sample closely resembled the overall proportion of these adult populations in the county (45% African American; 55% white).

Table 1 presents demographic and baseline characteristics for the final sample consisting of 477 African American and 688 white adults. A series of *t* tests and chi-square tests (data not shown) indicated that the African American respondents were on average more likely to have lower household incomes and lower education levels than white respondents (*P* < .001). There were no significant group differences for sex, age, body mass index, PA level, or walking behavior. Both African American and white groups included more than 50% females, more than 60% categorized as overweight or obese, and more than 59% as not meeting recommended levels of either PA or walking.

### Associations between race and perceptions of social and environmental supports for PA and walking

From the final sample of both white and African American respondents, 6% to 11% reported that they did not know or were not sure if their neighbors could be trusted, and 8% to 11% reported that they did not know or were not sure if their neighbors were physically active. Nearly one fourth of both white (24%) and African American (23%) adults reported that there were no public recreation facilities in their neighborhoods.

Table 2 presents descriptive information on perceptions of social and safety-related environmental supports for African American and white adults. Compared with African American adults, a greater percentage of white adults reported that they trusted their neighbors (94.3% white, 80.1% African American), had light traffic volume in their neighborhoods (46.7% white, 34.1% African American), and had neighborhoods safe from crime (74.6% white, 65.0% African American). A greater percentage of African American adults (47.6%) reported moderate traffic in their neighborhood, compared with white adults (36.3%). White and African American adults reported similar perceptions of neighbors' PA level, safety of public recreation facilities, streetlight quality, and problems with unattended dogs.

A series of logistic regression models were performed to determine the associations between race and perceptions of social and safety-related environmental supports for PA and walking (Tables 3 and 4). Table 3 shows the results for perceptions of social and safety environmental support variables stratified by race for individuals who reported meeting and not meeting the CDC recommendation for PA. White adults who perceived their neighbors as physically active were twice (95% CI, 1.19–3.25) as likely to report meeting the recommendation for PA, compared with white adults who did not perceive their neighbors as physically active. There were no significant differences in perceptions of social and safety-related environmental supports between African American adults reporting meeting or not meeting PA recommendations.

The regression model for perceptions of social and safety-related environmental support variables stratified by race for respondents who reported meeting and not meeting walking recommendations is presented in Table 4. White adults who perceived their neighbors as active were 2.5 times (95% CI, 1.54–4.08) more likely to meet the walking recommendation than white adults who did not perceive their neighbors as active. White adults who reported their neighborhoods as safe were 1.8 times (95% CI, 1.03–3.12), more likely to report meeting the walking recommendation than white adults who reported their neighborhoods as not safe. In addition, white adults who perceived moderate traffic in their neighborhood were one half as likely to report meeting the walking recommendation compared with white adults who perceived heavy traffic in their neighborhood. However, there were no significant differences in perceptions of social and safety-related environmental supports between African American adults reporting meeting or not meeting walking recommendations.

## Discussion

Of particular interest are the findings from this study indicating that, after adjusting for age, education, and sex of the respondents, no differences in perceptions of social or safety-related environmental factors were found between African American adults who reported meeting the recommended level of PA or walking and African American adults who reported not meeting the recommendation. The lack of association is surprising because the factors included in this study — such as safety factors related to traffic and crime — have been previously mentioned in several focus groups by African American adults as influences on PA level (11-17). It has been suggested, however, that small group discussions such as focus groups may elicit interaction and more thought about a topic among participants than the objective-response choices in a quantitative survey (33). Inherent differences may exist between qualitative and quantitative research techniques; investigators need to consider these differences when constructing quantitative data-collection instruments based on findings from qualitative studies and when comparing results from investigations using these methods (29).

Previous cross-sectional studies have substantiated an association between perceptions of safety related to traffic and crime and other environmental factors (e.g., streetlight quality, unattended dogs, trust in neighbors) and PA and walking in African American and other adult populations, including people living in rural locations (23,28,30). However, some studies have failed to observe such associations (25). For example, Eyler et al (29) found few similarities in social and environmental correlates of PA within a sample of women from the same racial and ethnic group. An Australian study also reported that adults living in high-walkable and low-walkable neighborhoods did not differ in their perceptions of safety related to traffic or crime (34). The lack of consistent results across studies and within groups indicates further research in this area is needed to better understand the environmental factors associated with PA and walking in people of varying races, ethnicities, and geographic locations. Furthermore, to determine the relative importance to PA and walking, the strength of these associations needs to be clarified and contrasted with other variables (e.g., self-efficacy, perceived health, anticipated benefits) that are consistently related to PA and walking (4-6,24,27).

White adults who perceived their neighbors as physically active were more likely to report meeting recommendations for PA and walking than white adults not perceiving their neighbors as physically active. In addition, white adults who perceived less crime in their neighborhood were more likely to report meeting recommendations for walking than those who perceived more crime in the neighborhood. It is not clear why perceptions of these two social and safety-related environmental factors were more strongly associated with PA and walking in white rather than African American adults. It has been postulated that differences may exist between how African American and white adults perceive features of the social and safety environments in their neighborhoods and this may potentially account for such results (10,22,31). For instance, Wilson et al (22) discovered that objective crime data did not support the reported differences in perceived crime between adults categorized as low and high income. We have also found that African American adults are more accurately aware of the presence of crime than white adults (D. Wilson, unpublished data, 2005). In addition, Boslaugh et al (10) reported that African American adults perceived their neighborhoods as less safe and less pleasant for PA than did white adults regardless of the racial composition of the neighborhood. Future studies will be required to determine whether perceived or objectively measured environmental factors are most influential on PA and walking in specific racial and ethnic groups, as well as what factors mediate or moderate their influence on PA-related behaviors.

Another possible explanation may be that race and ethnicity are simply serving as proxies for differing levels and types of PA among African American and white adults. A large proportion of white adults report engaging in structured and planned PA whereas African American adults, particularly women, equate PA with being busy at home or work or caring for children (12,13,35). It has been suggested that active people may be more aware of their environment; this explains the stronger association between perceptions of environmental attributes and PA found among physically active persons (25), but this association would likely hold true only for people who are physically active outdoors. One could then speculate that white adults engage more often in outdoor PA than African American adults, thus contributing to differing perceptions of environmental attributes. It has also been reported that perceptions of environmental attributes differ across levels of PA (31,36) and walking (36). This should not have been a factor in the current study because there were no differences between proportions of white and African American adults meeting PA and walking recommendations. Age and sex have also been shown to influence the association between perceptions of environmental attributes and walking (36), but these variables, along with level of education, were controlled for in our analyses. However, African American adults may accumulate daily walking differently than white adults (i.e., at work or home rather than during leisure time) (12,35). This difference is potentially important because different environmental attributes have been associated with walking for different purposes (e.g., exercise, pleasure) (36) as well as PAs of differing intensity (27). Therefore, variations in preferences and purposes for PA and types of PA may create varying perceptions of social and safety-related environmental factors among people from different racial and ethnic groups, even if they live in the same neighborhood. Further studies exploring the precise meaning of PA among adults from different racial and ethnic groups and neighborhoods and studies using more objective and comprehensive measures of daily PA are needed to help clarify these issues.

One finding that seemed counterintuitive was that white adults who perceived moderate traffic in their neighborhood were about one half as likely to meet the recommendation for walking than white adults who perceived heavy traffic in their neighborhood. However, in agreement with the current results, other studies have also noted an unexpected association between perceptions of traffic safety and PA or walking (25,26,34). For example, Humpel et al (26) reported that Australian adults who perceived traffic as not being a problem were 55% less likely to walk in their neighborhood. While the direction of the association in the present study was not necessarily anticipated, the odds ratio results provide evidence that elements of traffic in neighborhoods may have varying degrees of influence on walking behavior in white adults. The relative strength of this influence may be questioned because there were no associations between perceived traffic level and PA among either white or African American adults or between perceived traffic level and walking among African American adults. Other studies (12,24,25,28,30) using qualitative and quantitative approaches have documented the potential impact of perceived traffic on PA; this environmental attribute deserves additional investigation.

Despite published studies (12,23,30,37,38) that cite inadequate streetlights, unattended dogs, and unsafe recreational facilities as deterrents, the current study found no association among African American or white adults between these factors and meeting PA or walking recommendations. In fact, both groups rated these neighborhood components favorably, with more than one half noting the presence of adequate streetlight quality, active neighbors, safe recreation facilities, and a lack of serious problems with unattended dogs. Therefore, in the present study, a relative lack of variability in the perception of these attributes by both African American and white adults may have limited the likelihood of finding a significant association between these attributes and PA or walking. The processes used in this study do not allow us to draw conclusions on whether the relative lack of variability was attributable to the design of the survey tool, strong similarities in environmental factors near respondents' homes, a combination of both, or other factors.

Limitations to the present study include the use of a cross-sectional design and self-reported measures, similar to limitations noted in recent reviews of studies investigating similar research questions (18-20,24,25). Although the survey response (54%) in this study was modest, the rate was consistent with previous studies using similar methodology (3,22,32), and the final study sample closely mirrored the racial and ethnic composition of the county. Generalization of the results may be somewhat limited because the survey was conducted during the winter in a predominantly rural county with several small towns and only one metropolitan area. Another potential limitation of the current study was that some measures demonstrated low to fair validity (? = ?0.02 to 0.28) (39). However, the "gold standard" objective indices for most perceptual constructs of the social and safety-related environmental attributes included in this and similar PA studies have not been identified or agreed upon (25,31). This is particularly true for the social environmental constructs of perceiving a neighbor as trustworthy or physically active; to our knowledge, these constructs have not been validated against any truly objective indices. In addition, the level of ? constituting adequate agreement between perceptions of environmental supports for PA and various objective measures of these supports has not been established. Researchers should continue to refine survey instruments and other data collection methods to be used in this line of research.  Indeed, additional work in this area is ongoing (40).

The factors included in this study also did not represent the full domain of possible social and safety-related or built environmental influences on PA (e.g., access, convenience, sidewalks, trails, aesthetics) (21,25,27).  Furthermore, concepts of social cohesion, social support, and trust are multifaceted and complex, and perhaps the extent of the influences of these factors on PA and walking among African American adults was not fully captured in our assessment (29). As noted earlier in this paper, PA behavior is impacted by many variables and disentangling the most influential perceptual or environmental factors is challenging (4,5). This is particularly true when considering the many types of people, PAs, locations, and circumstances that need to be included in a comprehensive research agenda or PA promotion program (6,25,40).

In summary, this study documented that white adults who reported perceiving their neighbors as being trustworthy and physically active were more likely to report meeting recommendations for PA or walking. No association between perceptions of neighborhood social and safety-related environmental factors and PA or walking was observed in African American adults. Further quantitative research is needed to identify perceptions of social and safety attributes of the neighborhood environment that strongly impact PA and walking levels of white and African American adults to build upon findings from qualitative research and to design effective interventions to promote PA.

## Figures and Tables

**Table 1:** Demographic Characteristics of African American and White Adult Telephone-Survey Respondents (N = 1165) in a Rural County in South Carolina , 2001

**Variables**	**% African American** **(n = 477)**	**% White** **(n = 688)**

**Sex**		

Male	38.2	47.2
Female	61.8	52.8

**Age, y **		

18-29	30.5	19.7
30-44	31.0	33.7
45-64	21.8	29.7
65-74	10.5	11.4
≥75	6.2	5.5

**Annual household income^a^ **		

<$25,000	59.0	26.7
$25,000-$50,000	29.2	40.4
>$50,000	11.8	32.9

**Education^a^ **		

<High school	20.0	8.7
High school graduate	37.9	29.7
Some college	26.3	34.5
College graduate	15.8	27.1

**Body mass index **		

<25.0 kg/m^2 ^(normal)	39.6	39.4
25.0-29.9 kg/m^2 ^(overweight)	31.5	37.7
≥30.0 kg/m^2 ^(obese)	28.9	22.9

**Meets CDC physical activity recommendation^b^**		

Yes	34.5	39.7
No	65.5	60.3

**Walks **≥**150 mins per wk **		

Yes	37.4	40.9
No	62.6	59.1

a White adult respondents were significantly different from African American adult respondents (P < .001).

b CDC indicates Centers for Disease Control and Prevention. Recommendation is ≥30 minutes per day for ≥5 days per week of moderate-intensity physical activity or ≥20 minutes per session for ≥3 days per week of vigorous-intensity physical activity.

**Table 2:** Perceptions of Social and Safety-related Environmental Supports Among African American and White Adult Telephone-Survey Respondents (N = 1165) in a Rural County in South Carolina, 2001

	**African American**		**White**	

	**No.**	**Weighted %**	**No.**	**Weighted %**

**Traffic in neighborhood**				

Heavy	110	18.3	140	17.0
Moderate	202	47.6	233	36.3
Light	165	34.1	312	46.7
Don’t know/not sure	0		3	

**Streetlight quality in neighborhood**				

Good	138	27.7	164	23.2
Fair	130	28.6	167	28.2
Poor	199	43.7	347	48.6
Don’t know/not sure	10		10	

**Unattended dogs in neighborhood**				

Big problem	170	34.7	227	30.0
Not much of a problem	123	29.3	189	30.9
Not a problem	178	36.0	262	39.1
Don’t know/not sure	6		10	

**Neighborhood safe from crime**				

Safe	304	65.0	500	74.6
Not safe	162	35.0	181	25.4
Don’t know/not sure	11		7	

**Neighbors can be trusted**				

Yes	341	80.1	607	94.3
No	83	19.9	40	5.7
Don’t know/not sure	53		41	

**Public recreation facilities are safe**				

Yes	311	87.8	478	92.0
No	57	12.2	48	8.0
None in community	109		162	

**Neighbors are physically active**				

Yes	306	71.2	402	65.1
No	117	28.8	231	34.9
Don’t know/not sure	54		55	

**Table 3:** Likelihood Among African American and White Adults of Meeting the CDC Physical Activity Recommendation^a^

**Variable**	**African American**		**White**	

	**OR (95% CI)^b^**	***P* value**	**OR (95% CI)**	***P* value**

**Traffic in neighborhood**		.72		.16

Light	0.75 (0.36-1.55)		1.77 (0.98-3.17)	
Moderate	0.95 (0.50-1.80)		1.21 (0.72-2.02)	
Heavy	1.00		1.00	

**Streetlight quality in neighborhood**		.06		.21

Good	1.24 (0.62-2.50)		1.64 (0.94-2.85)	
Fair	0.50 (0.25-1.01)		1.27 (0.72-2.22)	
Poor	1.00		1.00	

**Unattended dogs in neighborhood**		.80		.32

Big problem	0.93 (0.49-1.77)		0.67 (0.39-1.15)	
Not much of a problem	1.20 (0.56-2.54)		0.76 (0.43-1.33)	
Not a problem	1.00		1.00	

**Neighborhood safe from crime**		.36		.50

Safe	1.32 (0.73-2.38)		0.84 (0.50-1.41)	
Not Safe	1.00		1.00	

**Neighbors can be trusted**		.18		.31

Yes	1.73 (0.77-3.89)		0.64 (0.26-1.53)	
No	1.00		1.00	

**Public recreation facilities are safe**		.73		.97

Yes	1.17 (0.48-2.84)		1.02 (0.44-2.33)	
No	1.00		1.00	

**Neighbors are physically active**		.32		.009

Yes	1.38 (0.73-2.63)		1.96 (1.19-3.25)	
No	1.00		1.00	

a CDC indicates Centers for Disease Control and Prevention. Recommendation is ≥30 minutes per day for ≥5 days per week of moderate-intensity physical activity or ≥20 minutes per session for ≥3 days per week of vigorous-intensity physical activity. Values are adjusted for education, age, and sex.

b OR indicates odds ratio; CI, confidence interval.

**Table 4:** Likelihood Among African American and White Adults of Walking at Least 150 Minutes Per Week^a^

**Variable**	**African American**		**White**	

	**OR (95% CI)^b^**	***P* value**	**OR (95% CI)**	***P* value**

**Traffic in neighborhood**		.81		.002

Light	0.83 (0.40-1.71)		1.56 (0.86-2.82)	
Moderate	1.04 (0.56-1.94)		0.52 (0.31-0.87)	
Heavy	1.00		1.00	

**Streetlight quality in neighborhood**		.80		.78

Good	0.84 (0.43-1.64)		1.18 (0.69-2.01)	
Fair	0.82 (0.43-1.59)		1.17 (0.67-2.05)	
Poor	1.00		1.00	

**Unattended dogs in neighborhood**		.99		.64

Big problem	1.00 (0.54-1.86)		0.77 (0.45-1.33)	
Not much of a problem	0.98 (0.47-2.05)		0.90 (0.52-1.57)	
Not a problem	1.00		1.00	

**Neighborhood safe from crime**		.38		.04

Safe	0.78 (0.44-1.36)		1.79 (1.03-3.12)	
Not safe	1.00		1.00	

**Neighbors can be trusted**		.52		.07

Yes	0.80 (0.40-1.59)		0.48 (0.21-1.07)	
No	1.00		1.00	

**Public recreation facilities are safe**		.93		.78

Yes	1.04 (0.43-2.50)		0.88 (0.37-2.09)	
No	1.00		1.00	

**Neighbors are physically active**		.46		<.001

Yes	1.26 (0.68-2.34)		2.51 (1.54-4.08)	
No	1.00		1.00	

a Values are adjusted for education, age, and sex.

b OR indicates odds ratio; CI, confidence interval.
